# Synthesis and evaluation of modified siRNA molecules containing a novel glucose derivative†

**DOI:** 10.1039/d1ra00922b

**Published:** 2021-03-01

**Authors:** Lidya Salim, Eva Goss, Jean-Paul Desaulniers

**Affiliations:** University of Ontario Institute of Technology, Faculty of Science 2000 Simcoe Street North Oshawa ON L1G 0C5 Canada jean-paul.desaulniers@ontariotechu.ca; Synthose Inc. 50 Viceroy Road Unit 7 Concord ON L4K 3A7 Canada

## Abstract

Chemical modifications are critical for the development of safe and effective siRNAs for downstream applications. In this study, we report the synthesis of a novel glucose phosphoramidite, a triazole-linked to uracil at position one, for incorporation into oligonucleotides. Biological testing revealed that the glucose derivative at key positions within the sense or antisense strand can lead to potent gene-silencing activity, thus highlighting its tolerance in both sense and antisense positions. Furthermore, the A-form helical formation was maintained with this modification. Overall, placing the modification at the 3′ end and at key internal positions led to effective RNAi gene-silencing activity.

## Abstract 2

Chemical modifications are critical for the development of safe and effective siRNAs for downstream applications.

## Graphical abstract

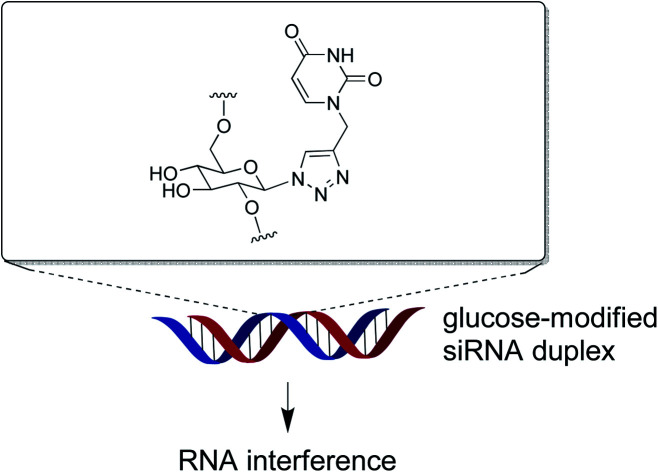

## Introduction

RNA interference (RNAi) is a natural mechanism that mediates sequence-specific gene silencing by targeting messenger RNA and suppressing translation.^1^ This pathway involves the assembly of an RNA-induced silencing complex (RISC) which incorporates double-stranded RNA sequences called short interfering RNAs (siRNAs).^2^ Each siRNA duplex is ∼21 nucleotides in length and is made up of a guide (antisense) strand and a passenger (sense) strand. After the siRNA duplex is unwound by RISC, the passenger strand is removed by the endonuclease Argonaute2 (Ago2), while the guide strand is retained and used as a guide sequence to locate and cleave the mRNA target.^3^ Synthetic siRNAs are compatible with the endogenous RNAi pathway and are able to reduce the expression of target proteins, serving not only as experimental tools but also as gene-silencing therapeutics. Despite recent advances in the field, such as the U.S. FDA approval of three RNAi-based therapies,^4,5^ the development of safe and effective siRNA therapeutics has been limited by the inherent structure of RNA which poses challenges like low stability, poor cellular uptake, and off-target effects.^6,7^

Chemical modifications can be used to optimize the pharmacokinetic properties of siRNAs for *in vivo* applications. Several modifications have been developed to date, including backbone, nucleobase, and sugar modifications, which can be incorporated individually or in combination.^8–10^ Nevertheless, there is still no universal modification that mitigates all the aforementioned challenges, so there is great interest in designing and investigating novel modifications that could be incorporated in for future siRNA design.

Modifications of the ribose sugar have been extensively studied to improve stability and siRNA potency. The presence of the 2′ hydroxyl group makes RNA more susceptible to hydrolysis and is often modified, as it is not required for RNAi activity.^11^ Common 2′ modifications include 2′-fluoro and 2′-methoxy, which increase siRNA stability.^12^ Other modifications include bicyclic derivatives like locked nucleic acids (LNA), which lock the ribose sugar in the 
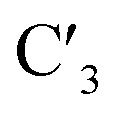
-*endo* conformation,^13^ and acyclic derivatives like unlocked nucleic acids (UNA), which lack the 
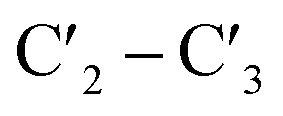
-bond of the ribose sugar.^14^

A more recent approach involves replacing the ribose sugar with six-carbon moieties. Altritol nucleic acids have displayed stronger activity than unmodified siRNAs, particularly when placed at the 3′ end of the sense or antisense strand.^15^ Cyclohexenyl and hexitol nucleic acids have also shown increased activity as well as nuclease stability.^16,17^ Herein, we explore the synthesis of a novel glucose phosphoramidite derivative, which is a triazole-linked to a uracil nucleobase at position one. This modification was introduced at either terminal or internal positions of the sense or antisense strand, resulting in siRNA duplexes containing a single 3′-6′/2′-5′ phosphodiester linkage.

## Experimental

### Chemicals and general methods

β-d-Glucopyranosyl azide was obtained from Synthose, Inc. Canada. Other starting reagents and solvents were obtained from other commercial sources such as Sigma Aldrich and used without further purification, unless otherwise stated. Standard flash chromatography was performed using Silicycle Siliaflash 60 (230–400 mesh). ^1^H, ^13^C and ^31^P NMRs were recorded in CDCl_3_ or CD_3_OD using a Bruker Avance III NMR spectrometer. NMR spectra are provided in the ESI Data.†

### Compound 1

To a solution of β-d-glucopyranosyl azide (0.5 g, 2.44 mmol) in anhydrous pyridine (7 mL) at 0 °C was added 1,3-dichloro-1,1,3,3-tetraisopropyldisiloxane (1.1 eq., 2.68 mmol, 0.86 mL). The mixture was allowed to equilibrate to room temperature and was stirred under argon for 6 hours. The reaction was quenched with methanol and concentrated *in vacuo*. The crude product was taken up in ethyl acetate and washed with water and sodium bicarbonate. The organic layer was dried over sodium sulphate, concentrated *in vacuo* and purified using flash chromatography (3 : 7 ethyl acetate/*n*-hexanes) to yield compound 1 as a white solid (0.677 g, 62%). ^1^H NMR (400 MHz, CDCl_3_). *δ* 4.59 (d, 1H), 4.10 (dd, 1H), 4.00 (dd, 1H), 3.82 (t, 1H), 3.6 (t, 1H), 3.33–3.28 (m, 2H), 1.11–1.02 (m, 28H). ^13^C NMR (101 MHz, CDCl_3_). *δ* 90.8, 78.7, 76.5, 73.4, 68.8, 60.6, 17.4, 17.3, 17.2, 17.1, 13.6, 13.2, 12.5.

### Compound 2

To a solution of compound 1 (0.45 g, 1 mmol) in DMF (5.5 mL) was added *p*-toluenesulfonic acid monohydrate (0.2 eq., 0.2 mmol, 0.038 g). The mixture was stirred at room temperature under argon. After 6.5 hours, the reaction mixture was diluted with ethyl acetate and washed with water and sodium bicarbonate. The organic layer was dried over sodium sulphate, concentrated *in vacuo* and purified using flash chromatography (3 : 7 ethyl acetate/*n*-hexanes) to yield compound 2 as a white solid (0.248 g, 55%).^1^H NMR (400 MHz, CDCl_3_). *δ* 4.62 (d, 1H), 3.95 (dd, 1H), 3.78 (dd, 1H), 3.73–3.66 (m, 2H), 3.48–3.44 (m, 1H), 3.39 (t, 1H), 2.62 (d, 1H), 1.12–1.02 (m, 28H). ^13^C NMR (101 MHz, CDCl_3_). *δ* 89.6, 79.8, 78.4, 73.8, 72.1, 61.9, 17.2, 12.9, 12.8, 12.1.

### Compound 3

To a solution of compound 2 (0.6 g, 1.34 mmol) in anhydrous pyridine (3 mL) was added anhydrous trimethylamine (0.56 mL, 4 mmol) under argon. While stirring the reaction at 0 °C, 4,4′-dimethoxytrityl chloride (1.5 eq., 2 mmol, 0.681 g) was added in 5 equal portions over a 5 hour period. The reaction mixture was allowed to equilibrate to room temperature and was stirred for an additional 7 hours. The solvent was removed *in vacuo* and the crude product was taken up in dichloromethane and washed with sodium bicarbonate. The organic layer was dried over sodium sulphate, concentrated *in vacuo* and purified using flash chromatography (3 : 7 ethyl acetate/*n*-hexane) to yield compound 3 as a yellow oil (0.75 g, 75%). ^1^H NMR (400 MHz, CDCl_3_). *δ* 7.22–7.08 (m, 9H), 6.75–6.73 (m, 4H), 4.5 (d, 1H), 3.85 (ddd, 1H), 3.7 (s, 6H), 3.7–3.64 (m, 1H), 3.61–3.58 (m, 1H), 3.38–3.34 (m, 1H), 3.31–3.27 (td, 1H), 2.6 (d, 1H), 1.03–0.93 (m, 28H). ^13^C NMR (101 MHz, CDCl_3_). *δ* 158.6, 147.3, 139.5, 129.1, 127.8, 127.1, 113.6, 113.2, 112.6, 89.6, 79.8, 73.9, 72.1, 61.9, 60.4, 17.2, 12.8, 12.1.

### Compound 4

To a mixture of compound 3 (0.25 g, 0.33 mmol) and propargyl uracil (0.055 g, 0.37 mmol) in anhydrous acetonitrile (5 mL) was added copper(i) iodide (0.007 g, 0.036 mmol) under argon. The solution was stirred at room temperature for 6 hours. The solvent was removed *in vacuo* and the crude product was purified using flash chromatography (gradient: 0% to 5% methanol/dichloromethane) to yield compound 4 as an off-yellow foam (0.23 g, 77%). ^1^H NMR (400 MHz, CD_3_OD). *δ* 9.10 (s, 1H), 7.52 (d, 1H), 7.46 (d, 1H), 7.36–7.31 (m, 5H), 7.25–7.19 (m, 4H), 7.10 (d, 1H), 6.87–6.84 (m, 4H), 5.73–5.64 (m, 1H), 5.0 (s, 1H), 4.10 (t, 1H), 3.97–3.87 (m, 2H), 3.72–3.63 (m, 2H), 3.57 (d, 1H), 2.8 (brs, 1H), 1.33–1.03 (m, 28H). ^13^C NMR (101 MHz, CDCl_3_). *δ* 158.6, 158.4, 147.3, 144.9, 139.5, 130.1, 129.1, 127.9, 127.8, 127.1, 113.6, 113.2, 102.8, 87.26, 86.44, 81.4, 79.9, 72.9, 71.9, 61.6, 51.9, 29.7, 17.3, 12.8, 12.1. ESI-HRMS (ES+) *m*/*z* calculated for C_46_H_61_N_5_O_10_Si_2_ + H^+^: 900.3957, found 900.4038 [M + H^+^].

### Compound 5

To a flame-dried round-bottomed flask was added a solution of compound 4 (0.25 g, 0.29 mmol) in anhydrous dichloromethane (4 mL), followed by the addition of anhydrous triethylamine (0.14 mL, 1.4 mmol) under an argon atmosphere. 2-Cyanoethyl-*N*,*N*-diisopropylchlorophosphoramidite (0.19 mL, 0.833 mmol) was then added drop-wise and the reaction was stirred at room temperature for 1.5 hours. Due to stability concerns, the crude product was purified using a short flash chromatography column (gradient: 20% to 70% ethyl acetate/*n*-hexane, maintaining 5% triethylamine) to yield compound 5 as a yellow oil (0.26 g, 84%), which was immediately used for solid-phase oligonucleotide synthesis. ^31^P NMR (162 MHz, CDCl_3_). *δ* ppm 147.83, 147.79.

### Oligonucleotide synthesis

Oligonucleotides were synthesized on an Applied Biosystems 394 DNA/RNA synthesizer using 1.0 μM controlled-pore glass (CPG) support columns and a 1.0 μM cycle with a 999 second coupling time. Phosphoramidites were resuspended in anhydrous acetonitrile, immediately before use, to a final concentration of 0.1 M. Oligonucleotide cleavage from the solid support columns was achieved by flushing the CPG columns with 1 mL EMAM solution (1 : 1 methylamine 33 wt% in ethanol/methylamine 40 wt% in water) for 1 hour at room temperature, followed by overnight incubation in EMAM to deprotect the bases. Oligonucleotides were concentrated in a MiVac Quattro Concentrator and later resuspended in DMSO (100 μM). The silyl protecting groups were removed by incubating the oligonucleotides with 3HF–Et_3_N (125 μL) for 3 hours at 65 °C. Crude oligonucleotides were precipitated in ethanol and desalted using Millipore Ampicon Ultra 3000 MW cellulose centrifugal filters. Strands were purified using reverse-phase HPLC eluting from 5% to 95% ACN in 0.1 M TEAA buffer (pH 7.0).

### Thermal denaturation and circular dichroism (CD) studies

For duplex formation, equimolar amounts of the respective sense and antisense strands were combined, dried down and resuspended in 400 μL sodium phosphate buffer (90 mM NaCl, 10 mM Na_2_HPO_4_, 1 mM EDTA; pH 7.0). Samples were heated for 2 minutes at 90 °C and allowed to slowly equilibrate to room temperature. Thermal denaturation and CD studies were performed using a Jasco J-815 CD Spectropolarimeter equipped with a temperature controller. To determine the melting temperature (*T*_m_) of each duplex, the change in absorbance at 260 nm was measured against a temperature gradient from 15 to 95 °C, at 0.5 °C min^−1^. Data were analysed using Meltwin v3.5 software. CD spectra were recorded at 25 °C, scanning from 200 to 40 nm with a screening rate of 20.0 nm min^−1^ and a 0.20 nm data pitch. Scans were performed in triplicate and averaged using Jasco's Spectra Manager v2 software.

### Biological assays

#### Cell culture and transfection

HeLa cells were maintained in Dulbecco's Modified Eagle's Medium (DMEM) supplemented with 10% fetal bovine serum (FBS) and 1% penicillin-streptomycin (Sigma). Cells were maintained at 37 °C in a humidified atmosphere with 5% CO_2_ and were passaged at 80% confluency. HeLa cells were seeded into 24-well plates, containing 400 μL DMEM (10% FBS), at a density of 5.0 × 10^4^ cells per well. Cells were incubated for 24 hours at 37 °C in a humidified atmosphere with 5% CO_2_ after which the culture medium was removed. For each transfection sample, a mixture of 1 μL Lipofectamine 2000™ (Invitrogen) and 49 μL 1X Gibco's Opti-MEM Reduced Serum Medium (Invitrogen) was incubated at room temperature for 5 min. Each siRNA was diluted in 1X Gibco's Opti-MEM Reduced Serum Medium on ice and mixed with 200 ng pGL3 and 50 ng pRLSV40 plasmids to achieve a final volume of 50 μL. The siRNA-plasmid mix was added to the Lipofectamine 2000™ Opti-MEM mix and incubated for 40 minutes at room temperature. These samples were then transferred to the respective wells of the 24-well plate and incubated for 24 hours at 37 °C prior to cell lysis.

#### Dual-Luciferase® Reporter Assay

Cells were lysed with 1× passive lysis buffer for 30 min at room temperature. Cell lysates (10 μL) were transferred to opaque Costar 96-well plates in triplicate for the Dual-Luciferase® Reporter Assay (Promega). Luciferase Assay Reagent II (LAR II) and Stop & Glo® Reagent were prepared following the manufacturer's protocol. LAR II (50 μL) was added to each well and luminescence was immediately measured using a Synergy HT (Bio-Tek) plate luminometer. Stop & Glo® (50 μL) was then added to each well and a second luminescence measurement was taken. Results are expressed as the ratio of firefly/*Renilla* luminescence taken as a percentage of an untreated control.

## Results and discussion

### Preparation of oligonucleotides

**Scheme 1:**
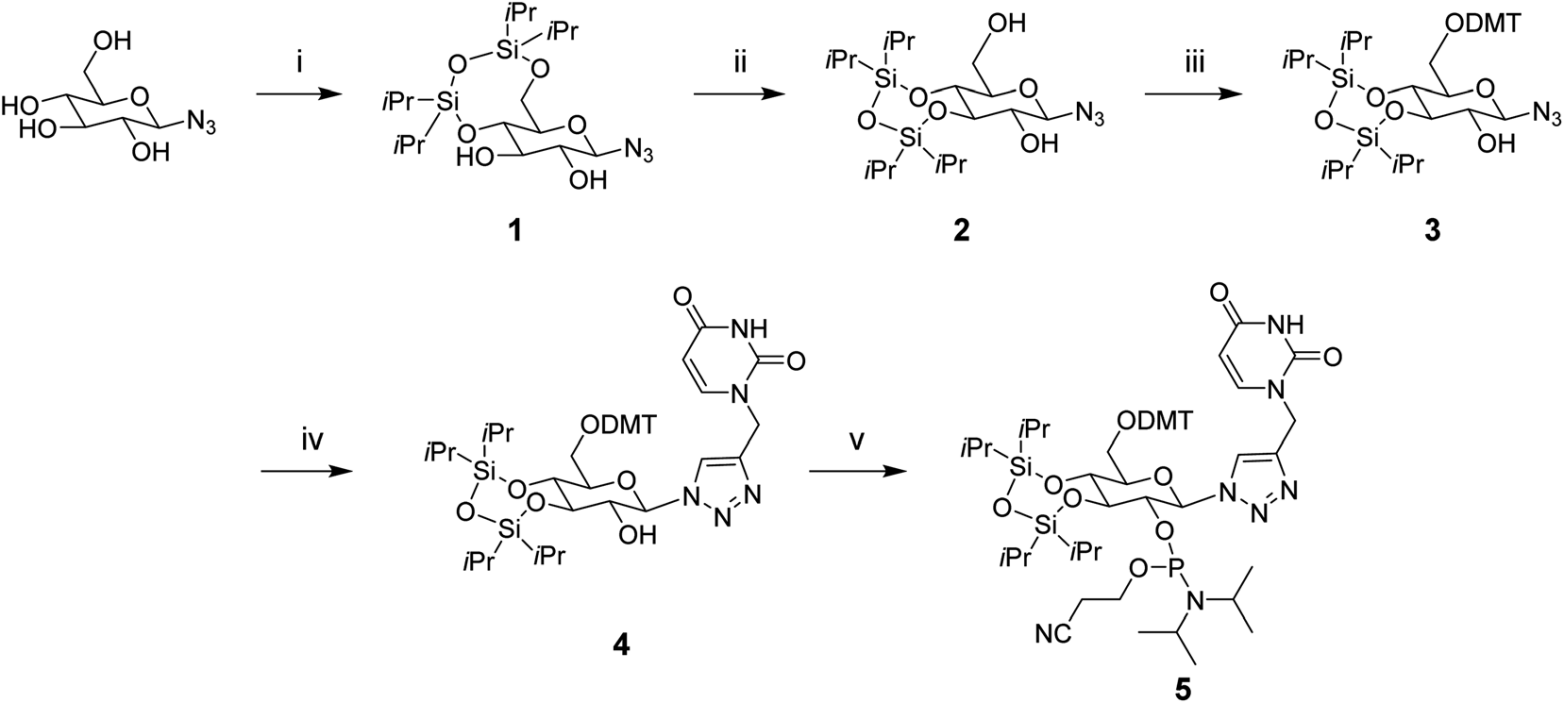
Synthesis of a glucose nucleoside containing a triazole-linked uracil base, and its phosphoramidite derivative. Reagents and conditions: (i) TIPDSCl_2_, pyridine, 0 °C → rt, 6 h (62%), (ii) *p*-TsOH·H_2_O, DMF, RT, 6.5 h (55%), (iii) DMT–Cl, Et_3_N/pyridine, 0 °C, 5 h, 0 °C → rt, 7 h (75%), (iv) *N*1-propargyl uracil, CuI, ACN, rt, 6 h (77%), (v) 2-cyanoethyl-*N*,*N*-diisopropylchlorophosphoramidite, Et_3_N/DCM, rt, 1.5 h (84%).

To synthesize the glucose phosphoramidite 5, we first treated β-d-glucopyranosyl azide with TIPDSCl_2_. This was followed by the acid-catalyzed migration of the 4,6-TIPDS protecting group to yield the 3,4-protected derivative 2, as previously reported in the literature.^18^ This compound was protected with 4,4′-dimethoxytrityl (DMT) and then reacted with *N*1-propargyl uracil *via* copper(i)-catalyzed azide–alkyne cycloaddition (CuAAC). The resulting compound 4 was phosphitylated with 2-cyanoethyl-*N*,*N*-diisopropylchlorophosphoramidite to yield the phosphoramidite derivative 5 (Scheme 1), which was used for solid-phase oligonucleotide synthesis as described above. The modification was incorporated at key positions within the sense or antisense strand, replacing either the 3′ dTdT overhang or an internal uridine nucleotide. Oligonucleotides strands were purified using reverse-phase HPLC (ESI Fig. S1†) and characterized by mass spectrometry (ESI Table S1†).

### CD studies

**Fig. 1:**
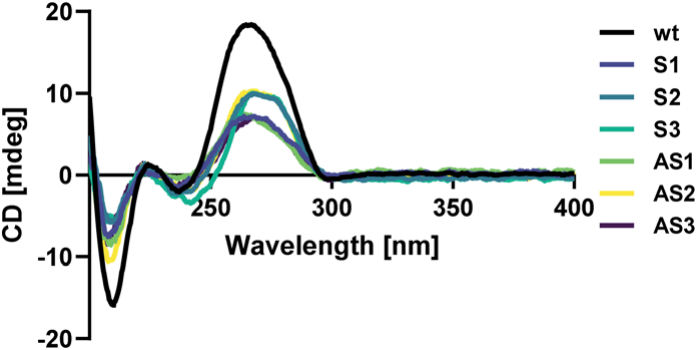
Circular dichroism (CD) spectra of anti-luciferase siRNAs.

Modified sense and antisense strands were annealed to their complementary wild-type sequences. The resulting duplexes were characterized using circular dichroism spectroscopy as described above to confirm that siRNAs adopted an A-form helical conformation. Recognition of the A-form major groove by RISC is required for proper RNAi activity, so this is an important criterion in siRNA design.^19^ An A-form helical structure is characterized by a broad positive band at 260 nm in addition to a negative band at ∼210 nm.^20^ As seen in Fig. 1, our modification did not distort the A-form helical structure of the siRNA duplex, regardless of its placement in the sequence.

### Thermal denaturation

Since the thermodynamic properties of siRNAs have been shown to impact siRNA potency, we assessed the thermal stability of each duplex. The resulting melting temperatures (*T*_m_) are indicated in Table 1. Placing our modification at the 3′ end of the sense or antisense strand, replacing the dTdT overhang, had a small impact on thermal stability with Δ*T*_m_ values of −5 °C. This could be due to the loss of stacking interactions which have been reported with 3′ dTdT overhangs.^21^ Internal modifications resulted in strong thermal destabilization.

**Table 1:** Sequences, melting temperatures and IC_50_ values of anti-firefly luciferase siRNAs^a^

Code	Duplex	*T*_m_ (°C)	Δ*T*_m_ (°C)	IC_50_ (pM)
wt	5′ CUU ACG CUG AGU ACU UCG ATT 3′	76.1	—	1.90
	3′ TTG AAU GCG ACU CAU GAA GCU 5′			
S1	5′ CUU ACG CUG AGU ACU UCG AX̲ 3′	71.1	−5.0	218
	3′ TTG AAU GCG ACU CAU GAA GCU 5′			
S2	5′ CUU ACG CUG AGU ACU X̲CG ATT 3′	50.1	−26.0	219
	3′ TTG AAU GCG ACU CAU GAA GCU 5′			
S3	5′ CUU ACG CUG AGX̲ ACU UCG ATT 3′	54.1	−22.0	524
	3′ TTG AAU GCG ACU CAU GAA GCU 5′			
AS1	5′ CUU ACG CUG AGU ACU UCG ATT 3′	71.1	−5.0	226
	3′ X̲G AAU GCG ACU CAU GAA GCU 5′			
AS2	5′ CUU ACG CUG AGU ACU UCG ATT 3′	59.1	−17.0	219
	3′ TTG AAX̲ GCG ACU CAU GAA GCU 5′			
AS3	5′ CUU ACG CUG AGU ACU UCG ATT 3′	53.6	−22.5	483
	3′ TTG AAU GCG ACX̲ CAU GAA GCU 5′			

a The top strand corresponds to the sense strand. The bottom strand corresponds to the antisense strand. X̲ corresponds to the triazole-linked uracil modification. Inhibitory dose–response curves can be found in the ESI Data (ESI Fig. S2).

Placing the modification at positions 12 or 16 from the sense strand 5′ end resulted in Δ*T*_m_ values of −22 °C and −26 °C, respectively. Similar effects were observed when placing the modification at positions 10 and 16 from the antisense strand 5′ end, with Δ*T*_m_ values of −22.5 °C and −17 °C, respectively. These results were expected as the internal region of siRNA is far less tolerant to bulky chemical modifications than the 3′ end.^22^

### Gene-silencing activity

**Fig. 2:**
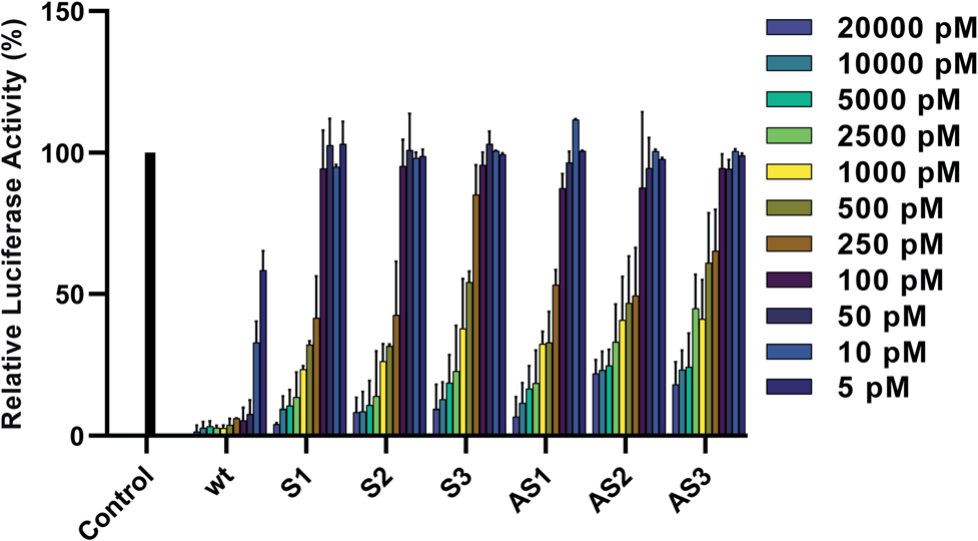
Relative expression of normalized firefly luciferase in HeLa cells 24 hours after siRNA treatment. Error bars indicate standard deviation of at least two independent biological replicates.

To assess the gene-silencing activity of siRNAs, HeLa cells were co-transfected with plasmids coding for firefly and *Renilla* luciferases as well as siRNAs, using Lipofectamine 2000™ (Invitrogen). We then used the Dual-Luciferase® Reporter Assay to evaluate the relative expression of target firefly luciferase after siRNA treatments ranging from 5 to 20 000 pM. As seen in Fig. 2, all tested siRNAs showed dose-dependent knockdown of firefly luciferase after 24 hours. IC_50_ values are summarized in Table 1. Duplexes bearing terminal modifications, placed at the 3′ end of the sense or antisense strand, showed high gene-silencing activity with IC_50_ values of 218 pM and 226 pM, respectively. This is consistent with literature reports showing that six-carbon sugar derivatives are well-tolerated and can lead to strong gene-silencing activity when placed at the 3′ end of the siRNA sense or antisense strand. Although internal modifications were tolerated in both the sense and the antisense strand, their effect on siRNA activity was position dependent. Placing our modification at position 16 from the sense or antisense strand 5′ end led to efficient gene-silencing activity (IC_50_ of 219 pM), comparable to our terminal-modified siRNAs. On the other hand, placing our modification at position 10 from the antisense strand 5′ end led to a decrease in gene-silencing activity (IC_50_ of 483 pM). It has been reported that the seed region, which directs the initial target recognition by RISC, is more sensitive to chemical modifications, particularly if they disrupt the thermal stability of the duplex.^22^ The lowest activity, however, was observed with siRNA S3, bearing the modification at position 12 from the sense strand 5′ end (IC_50_ of 524 pM).

Some reports suggest that this position can be less tolerant to chemical modifications, including altritol nucleic acids.^23^ Given the proximity to the Ago2 cleavage site, it has been proposed that some chemical modifications at this position can interfere with the enzymatic activity of Ago2 thus compromising siRNA potency.^24^ Based on these data, this modification may be better suited for incorporation at the 3′ end of the sense or antisense strand as well as at some internal in order to maximize gene-silencing activity.

## Conclusion

In summary, we report the synthesis of a novel glucose phosphoramidite with a triazole-linked uracil moiety at position 1 for incorporation into oligonucleotides using standard solid-phase synthetic conditions. This modification was placed at terminal and internal positions of the siRNA sense or antisense strand to investigate its biophysical and biological effects. Overall, this modification was well-tolerated within the sense and the antisense strand and did not distort the A-form helical conformation of the siRNAs, making it suitable for RNAi applications. Notably, our modified siRNAs show position-dependent gene-silencing activity. Replacing the dT overhang at the 3′ end or modifying position 16 from the 5′ end of either stand resulted in high siRNA activity. This position-dependent effect could be further investigated to optimize siRNA potency. Although there are some general guidelines for siRNA design, these criteria are not universally applicable, highlighting the importance of assessing the effect of each chemical modification individually. To the best of our knowledge, this is the first report of an siRNA bearing a single 3′-6′/2′-5′ phosphodiester linkage.

## Author contributions

Lidya Salim: data curation; investigation; methodology; visualization; writing – original draft. Eva Goss: funding acquisition; project administration; resources. Jean-Paul Desaulniers: conceptualization; funding acquisition; project administration; resources; supervision; writing – review & editing.

## Conflicts of interest

There are no conflicts to declare.

## Supplementary Material

RA-011-D1RA00922B-s001
