# Supplementary material for: Synthesis and evaluation of modified siRNA molecules containing a novel glucose derivative
Source: RSC Adv. 2021 Mar 1;11(16):9285–9. doi: 10.1039/d1ra00922b (PMC8698894; doi:10.1039/d1ra00922b)

Electronic Supplementary Material (ESI) for RSC Advances.
This journal is © The Royal Society of Chemistry 2021

Supplementary Data

Synthesis and Evaluation of Modified siRNA Molecules Containing a Novel Glucose Derivative

Lidya Salim,[1] Eva Goss[2] and Jean-Paul Desaulniers[1]

[1]Faculty of Science, University of Ontario Institute of Technology, Faculty of Science, 2000 Simcoe Street North, Oshawa, ON L1G 0C5 Canada

[2]Synthose, Inc. 50 Viceroy Road, Unit 7, Concord, ON L4K 3A7 Canada

Procedures

Procedure for LC/MS……S2

Tables

Table S1. Sequences and mass spectrometry data of modified oligonucleotide strands……S3

Figures

Figure S1. Analytical HPLC traces of modified oligonucleotides……S4

Figure S2. Inhibitory dose-response curves for modified anti-luciferase siRNAs……S4

NMR spectra

$^{1}H/^{13}C$ NMR Spectra of Compound 1……S5

$^{1}H/^{13}C$ NMR Spectra of Compound 2……S6

$^{1}H/^{13}C$ NMR Spectra of Compound 3……S7

$^{1}H/^{13}C$ NMR Spectra of Compound 4……S8

^{31}P NMR Spectrum of Compound 5……S9

Procedures

Procedure for LC/MS: LC/MS chromatograms were acquired on an Agilent 6545 QTOF-MS with Agilent 1260 Infinity Binary Pump HPLC using a ZORBAX Eclipse Plus C18 2.1x100mm 1.8-Micron Agilent column and a mobile phase of 5 mM ammonium acetate buffer (pH 7)/acetonitrile (95:5). Oligonucleotide samples were prepared at a concentration of 0.01 O.D/µL with an injection volume of 20 µL. Data were analysed using Agilent Technologies MassHunter Workstation Qualitative Analysis Software (Qual. 10.0).

Tables

Table S1. Sequences and mass spectrometry data of modified oligonucleotide strands

Code	*Sequence*	*Mass (predicted)*	*Mass (found)*
S1	5’ CUU ACG CUG AGU ACU UCG A**X** 3’ (S)	6796.88	6796.52
S2	5’ CUU ACG CUG AGU ACU **X**CG ATT 3’ (S)	6794.90	6795.56
S3	5’ CUU ACG CUG AG**X** ACU UCG ATT 3’ (S)	6794.90	6794.62
AS1	3’ **X**G AAU GCG ACU CAU GAA GCU 5’ (AS)	6882.94	6882.87
AS2	3’ TTG AA**X** GCG ACU CAU GAA GCU 5’ (AS)	6880.96	6880.80
AS3	3’ TTG AAU GCG AC**X** CAU GAA GCU 5’ (AS)	6880.96	6880.61

(S) corresponds to the sense strand; (AS) corresponds to the antisense strand. **X** corresponds to the position of the glucose nucleoside with a triazole-linked uracil.

Figures

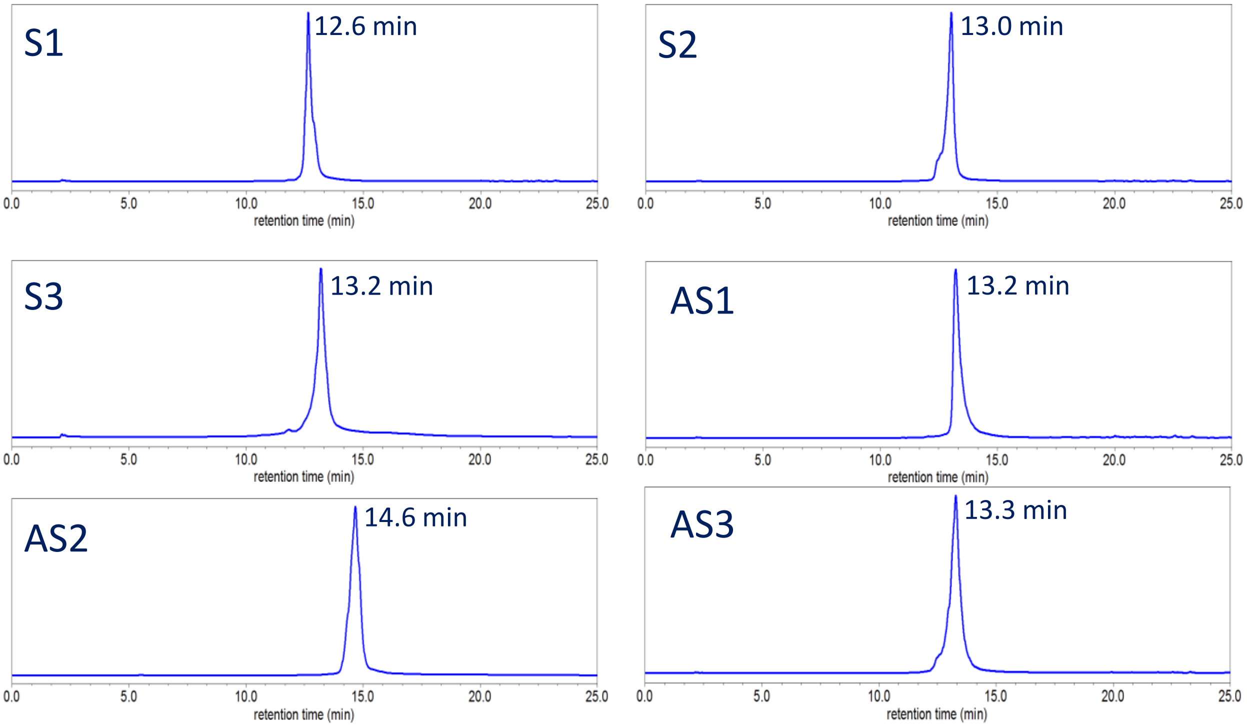

Figure S1. Analytical HPLC traces of modified oligonucleotides. Corresponding sequences can be found in Table S1. HPLC was performed on a Waters 1525 binary HPLC pump with a Waters 2489 UV/Vis detector, using a C18 4.6 x 150 mm reverse-phase column, eluting from 5 to 95% ACN in 0.1 M TEAA buffer (pH 7.0).

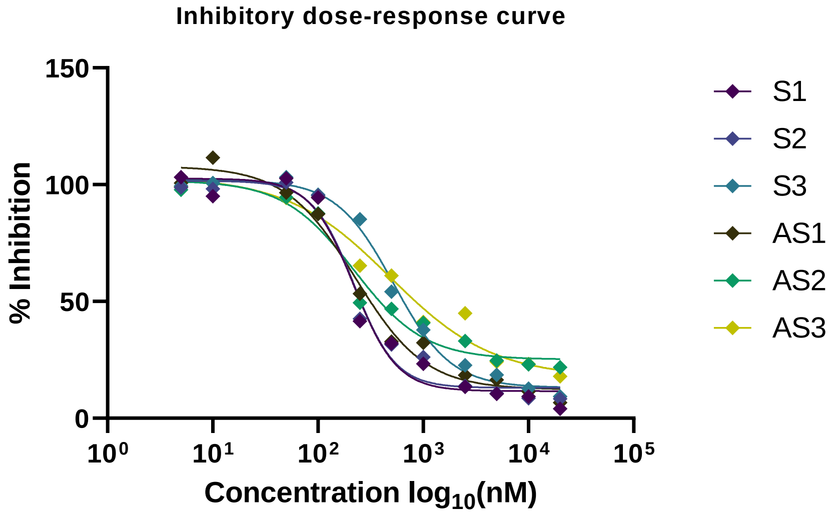

Figure S2. Inhibitory dose-response curves for modified anti-luciferase siRNAs, tested in HeLa at concentrations from 5 to 20,000 pM.

NMR Spectra

^{1}H NMR Spectrum of Compound **1**

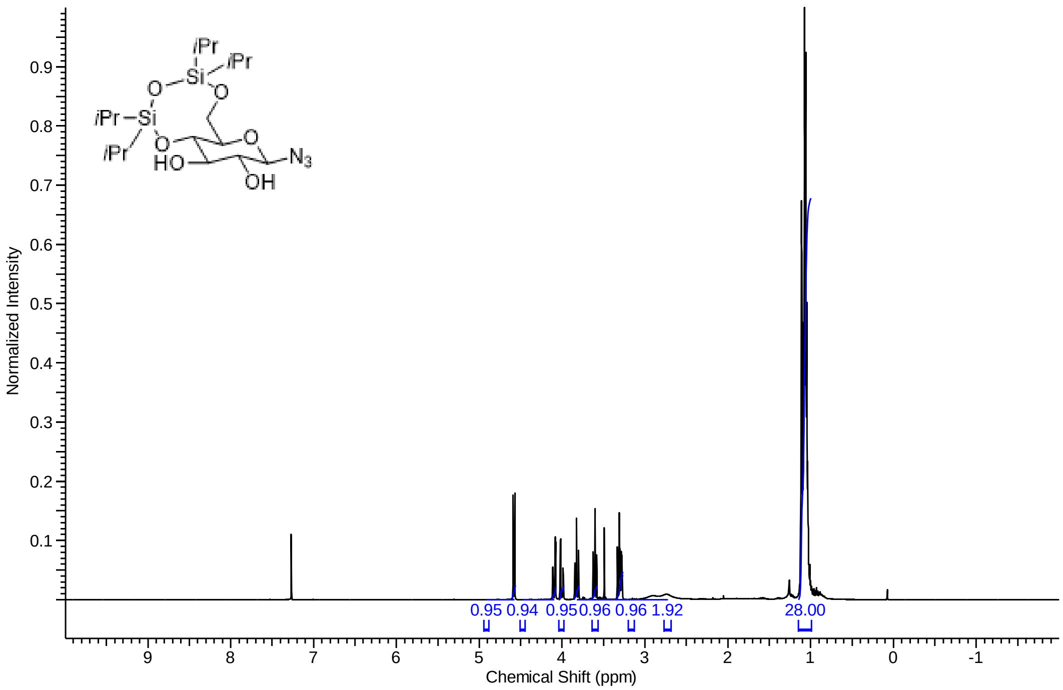

^{13}C NMR Spectrum of Compound **1**

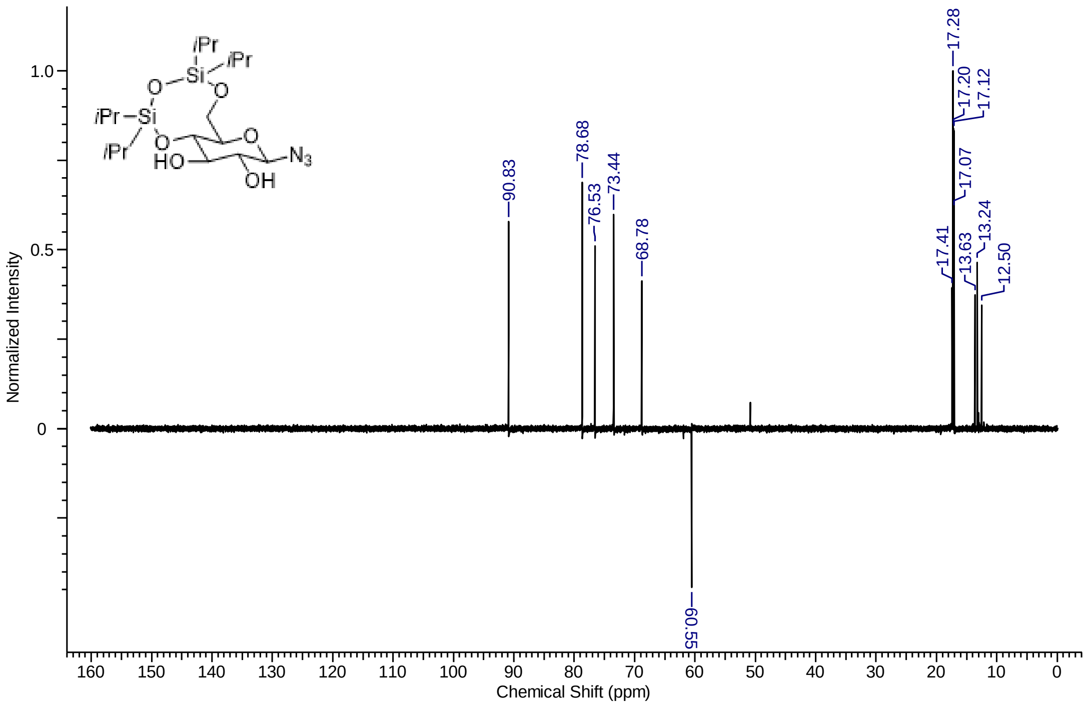

^{1}H NMR Spectrum of Compound **2**

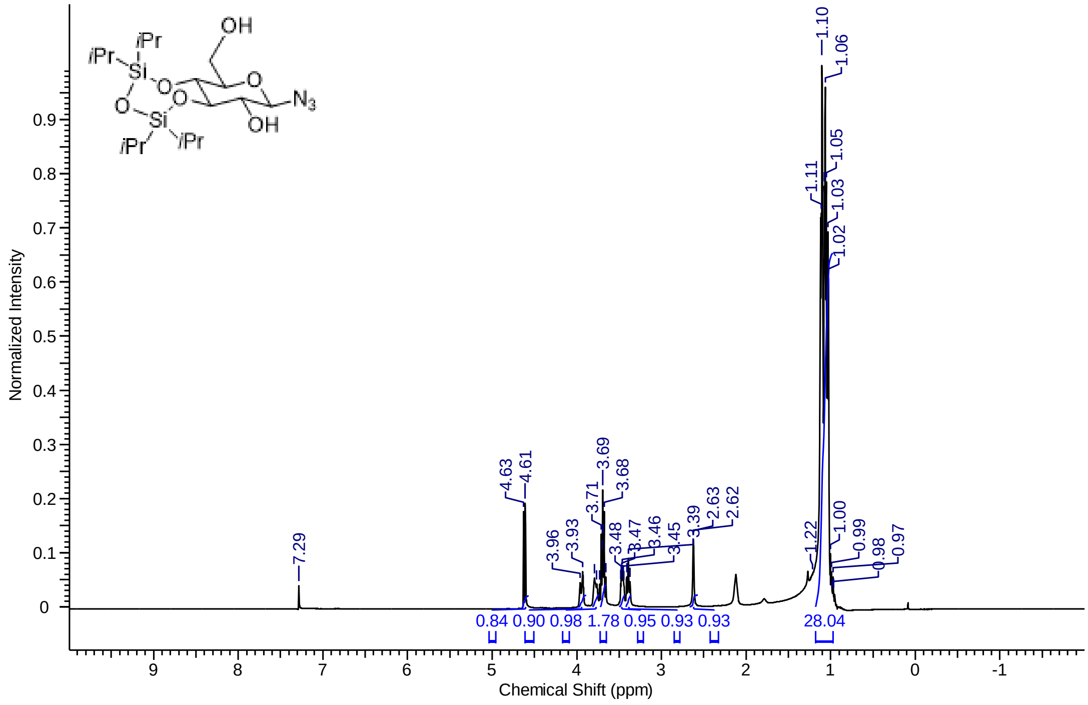

^{13}C NMR Spectrum of Compound **2**

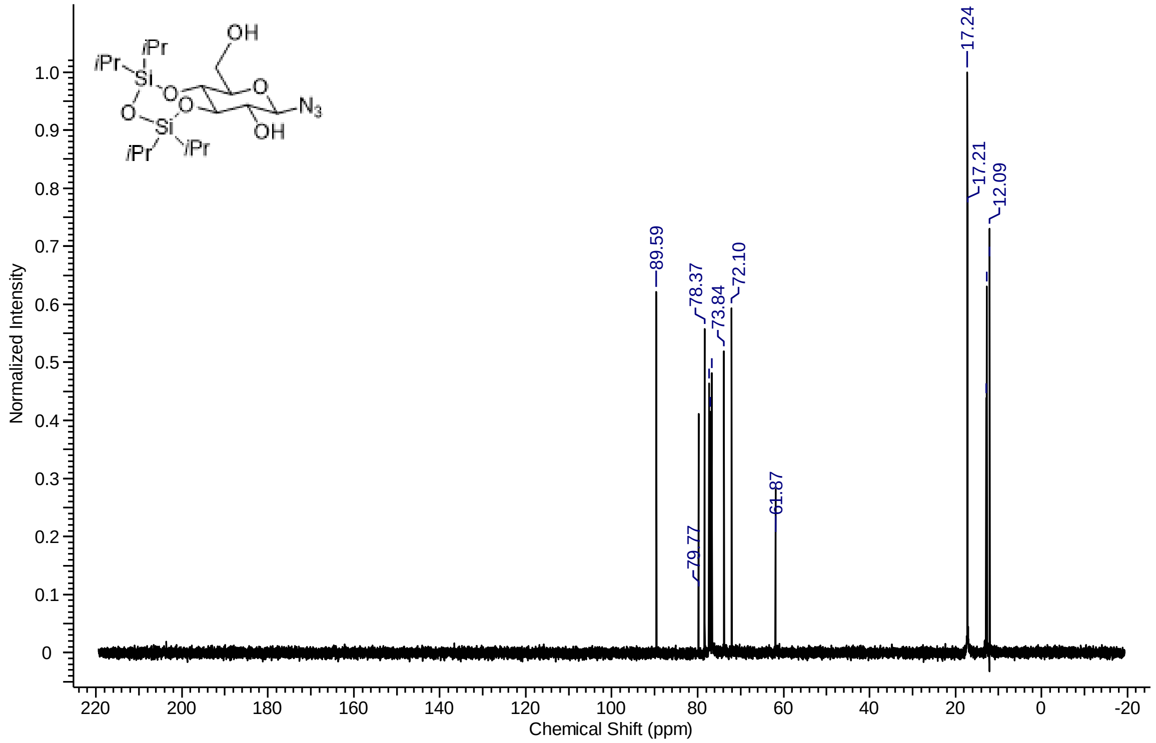

^{1}H NMR Spectrum of Compound **3**

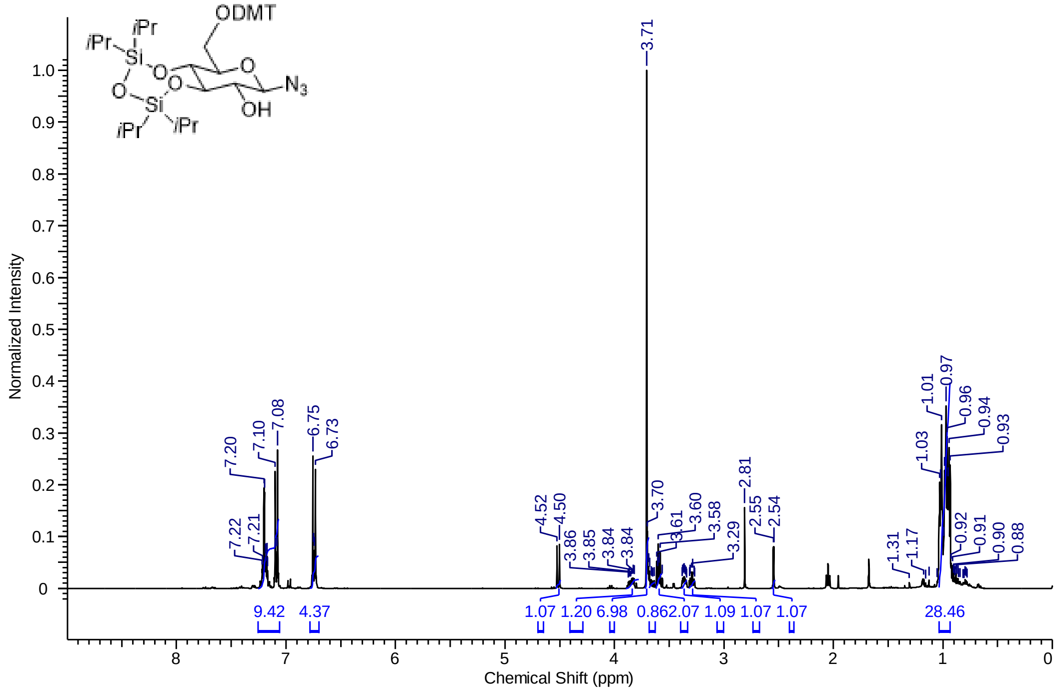

^{13}C NMR Spectrum of Compound **3**

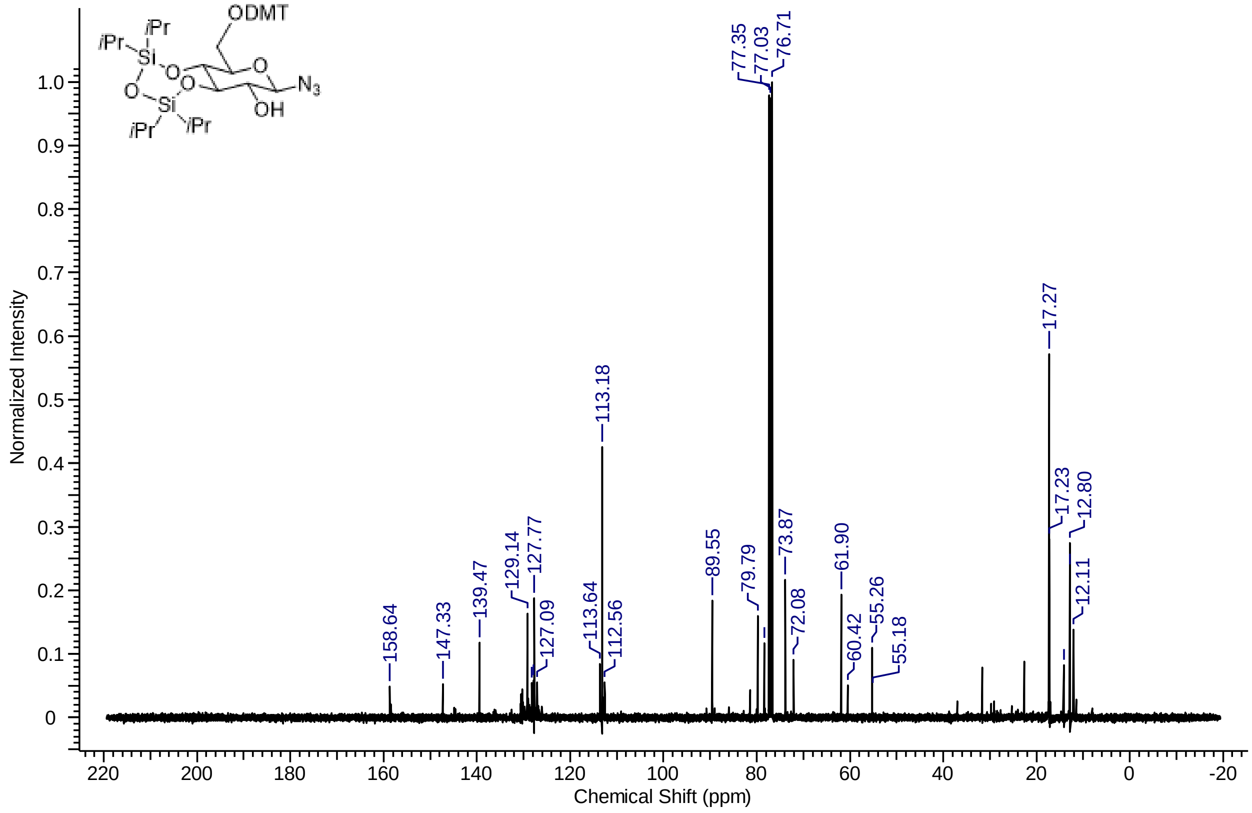

^{1}H NMR Spectrum of Compound **4**

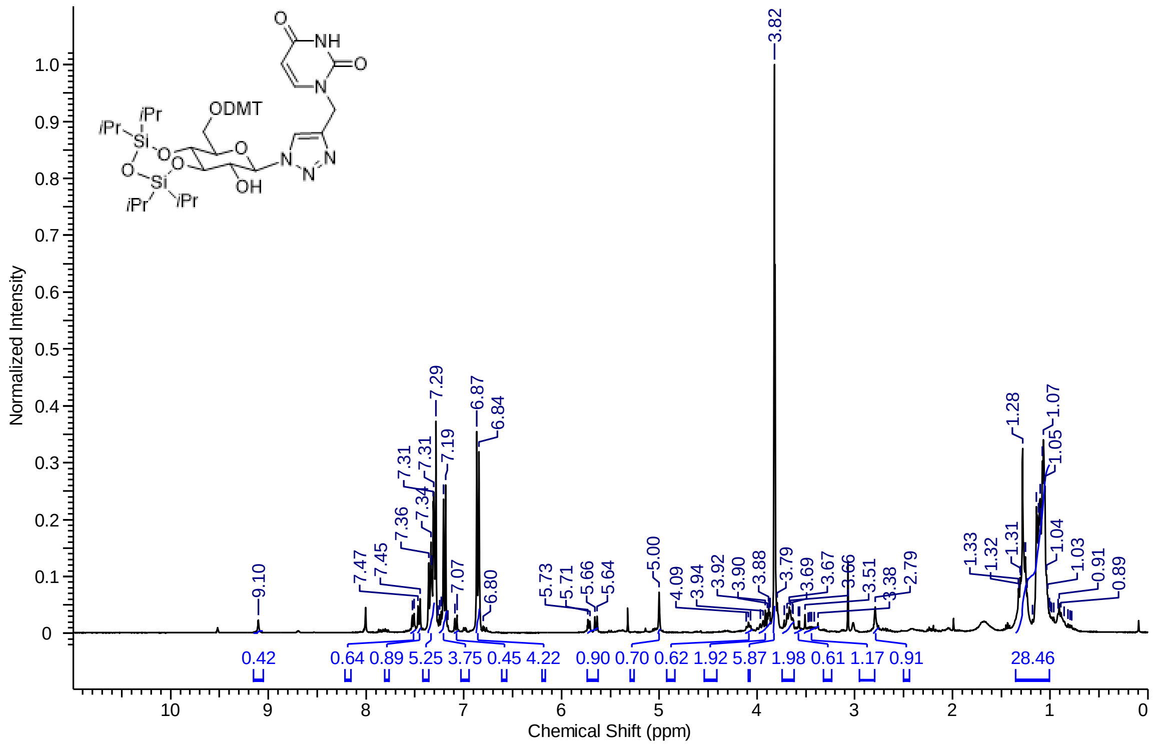

^{13}C NMR Spectrum of Compound **4**

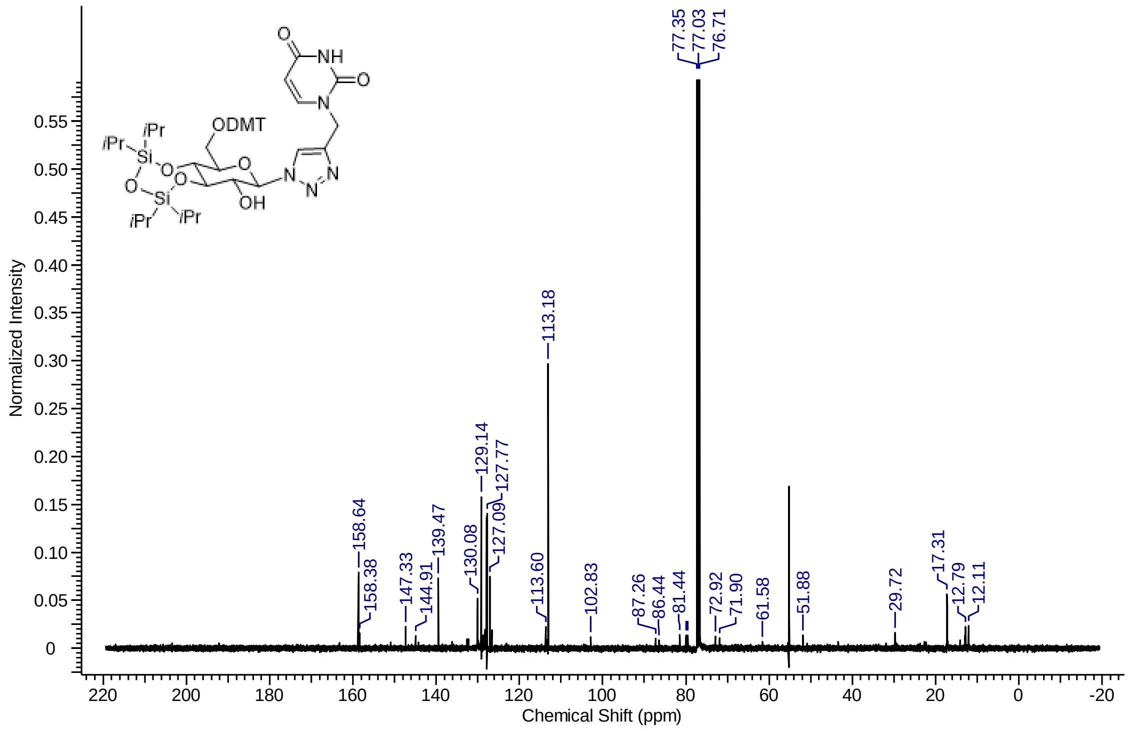

^{31}P NMR Spectrum of Compound **5**

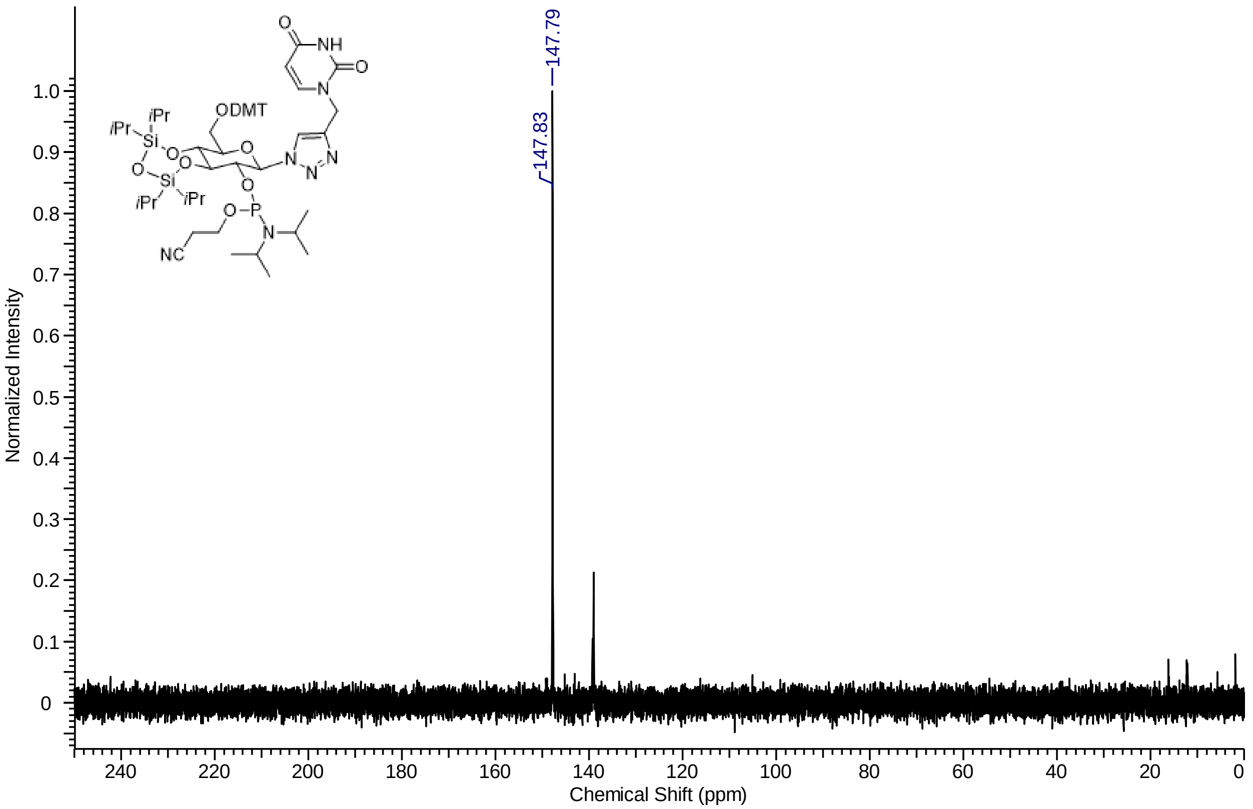

Supplement: RA-011-D1RA00922B-s001 [file RA-011-D1RA00922B-s001.pdf]
